# Gleanings from European Journals

**Published:** 1889-09

**Authors:** 


### Translations.

### GLEANINGS FROM EUROPEAN JOURNALS.

In *Le Progrès Médical* of July 13th, Prof. Althaus, of London, describes a contrivance which he has added to the Sayre apparatus, enabling him to improve upon the old method of suspending patients afflicted with locomotor ataxia, etc.

It consists of a crank and handle firmly attached to one of the legs of the tripod, by means of which he is able to raise his patients slowly and uniformly from the floor. The old method of simply pulling on the cord subjected the patient to much unpleasantness and inconvenience, in being elevated by a series of jerks. The new method is certainly more agreeable to the one undergoing treatment, and less arduous and tiresome to the physician in charge.

Prof. Althaus sums up his experience with suspension in the following words:

> I have now made about 450 suspensions on thirty-eight individuals, and, although it is somewhat premature to pronounce definitely upon the subject, nevertheless I am convinced that the suspension method will remain a valuable agent in the treatment of chronic diseases of the nervous system. In some of my cases the results have been truly remarkable. One of my patients, suffering from locomotor ataxia for a number of years, has actually regained his patellar reflexes. The case is that of a man fifty-six years old, whom I have treated since 1885. As far as I am able to learn, it is the first case on record where the patellar reflexes, being absent for a number of years, have reappeared under the influence of suspension.
>
> In the majority of my cases the patients have improved; in a certain number, however, the results have been negative, or nearly so. I have followed this mode of treatment in locomotor ataxia, spastic spinal paralysis, amyotrophic lateral sclerosis, paralysis agitans, neurasthenia, chronic rheumatism, and rheumatic gout.

Profs. Mendel and Eulenburg, of Berlin, have recently published the results of their experience with this method in the *Neurologisches Centralblatt:*

> Forty patients were under treatment—thirty-one males, nine females. Of this number thirty-four were afflicted with locomotor ataxia, one with disseminated sclerosis, one with chronic myelitis, one with traumatic neurosis, and three with paralysis agitans.

Favorable results were obtained in the case of disseminated sclerosis, paralysis agitans, and in the majority of patients suffering with locomotor ataxia. Negative results were obtained in five cases of locomotor ataxia.

---

In the *Sociètè de Biologie* (Paris) of July 6, 1889, Prof. Magnan read a paper on the therapeutic action of chlorhydrate of hyoscine.

In concert with M. Lefort, M. Magnan has employed hyoscine in his service upon a large number of maniacs, and has found its action marvelous. Doses of one milligram calmed his patients in five minutes. No unpleasant effects were observed in its application, except a vaso-motor disturbance in two cases, and a slight syncope in a tubercular patient. The duration of its action varied from five to ten hours. In cases of mania, one obtains, therefore, with hyoscine, an immediate and continued repose, not possible by any other agent.

Prof. Magnan obtained a continued and prolonged sleep with hyoscine in acute alcoholic delirium. In a child with "tic convulsiv" of the face and limbs, the spasmodic movements were almost entirely controlled. The hyoscine employed was prepared by Landenburg.

---

DEPOPULATION IN FRANCE.—To combat against the increasing depopulation in France, the Chamber of Deputies, upon the motion of Dr. Jarval, has exempted all fathers or mothers of seven children or over, from payment of poll and personal taxes.

---

THE International Congress for Physiological Psychology took place at Paris, August 5th to 10th, under the presidency of Prof. Charcot.

---

THE new military law in France requires all medical students to serve only one year under the flag, provided they obtain their degree, Doctor of Medicine, before their twenty-sixth year. Failing to do this, they will be compelled, like other privates, to serve three years.

W. C. K.

---

THE WINE AGREED WITH THEM.—Young waiter (at a medical dinner)—"Them doctors use a lot of wine, but I s'pose they kin stand it."

Old waiter—"Dunno about that; I'm thinkin' they're gettin' pretty tight already."

"They don't look so."

"No; but they're beginnin' to agree."—*Philadelphia Record.*

